# Personalized Decisional Algorithms for Soft Tissue Defect Reconstruction after Abdominoperineal Resection for Low-Lying Rectal Cancers

**DOI:** 10.3390/curroncol31060247

**Published:** 2024-06-04

**Authors:** Dan Cristian Moraru, Mihaela Pertea, Stefana Luca, Valentin Bejan, Andrian Panuta, Raluca Tatar, Dan Mircea Enescu, Dragos Viorel Scripcariu, Viorel Scripcariu

**Affiliations:** 1 Department of Plastic Surgery, Faculty of Medicine, “Grigore T. Popa” University of Medicine and Pharmacy, 700115 Iași, Romania; cristian-dan.moraru@umfiasi.ro (D.C.M.); stefana_luca@umfiasi.ro (S.L.); 2 Plastic, Reconstructive Surgery and Burns Clinic, “Sf. Spiridon” Emergency County Hospital, 700111 Iași, Romania; andrian.s.panuta@umfiasi.ro; 3 Department of Surgery, Faculty of Medicine, “Grigore T. Popa” University of Medicine and Pharmacy, 700115 Iași, Romania; valentin.bejan@umfiasi.ro (V.B.); viorel.scripcariu@umfiasi.ro (V.S.); 4 First Surgery Clinic, “Sf. Spiridon” Emergency County Hospital, 700111 Iași, Romania; 5 Faculty of Medicine, “Carol Davila” University of Medicine and Pharmacy, 020021 Bucharest, Romania; raluca.tatar@umfcd.ro (R.T.); enescudanmircea@gmail.com (D.M.E.); 6 Department of Plastic, Reconstructive Surgery and Burns, “Grigore Alexandrescu” Clinical Emergency Hospital for Children, 011743 Bucharest, Romania; 7 First Oncological Surgery Clinic, Regional Institute of Oncology (IRO), 700483 Iași, Romania

**Keywords:** decisional algorithms, rectal cancer, abdominoperineal excision of the rectum, perineal defect, perineal reconstruction, direct closure, flaps

## Abstract

Background: Abdominoperineal resection (APR)—the standard surgical procedure for low-lying rectal cancer (LRC)—leads to significant perineal defects, posing considerable reconstruction challenges that, in selected cases, necessitate the use of plastic surgery techniques (flaps). Purpose: To develop valuable decision algorithms for choosing the appropriate surgical plan for the reconstruction of perineal defects. Methods: Our study included 245 LRC cases treated using APR. Guided by the few available publications in the field, we have designed several personalized decisional algorithms for managing perineal defects considering the following factors: preoperative radiotherapy, intraoperative position, surgical technique, perineal defect volume, and quality of tissues and perforators. The algorithms have been improved continuously during the entire period of our study based on the immediate and remote outcomes. Results: In 239 patients following APR, the direct closing procedure was performed versus 6 cases in which we used various types of flaps for perineal reconstruction. Perineal incisional hernia occurred in 12 patients (5.02%) with direct perineal wound closure versus in none of those reconstructed using flaps. Conclusion: The reduced rate of postoperative complications suggests the efficiency of the proposed decisional algorithms; however, more extended studies are required to categorize them as evidence-based management guide tools.

## 1. Introduction

Colorectal cancer is the most common digestive neoplasm; in Romania, it ranks second in incidence and mortality in both sexes, trending upwards in recent decades. Colorectal cancer was the third neoplasia after broncho-pulmonary and breast cancer, representing 10% of all cancers worldwide in 2021 [1,2]. The incidence of colorectal cancer according to GLOBOCAN 2020 statistics places it as the third most common neoplasia in men (16.9/21.1 per 100,000 for rectal/colon cancer) and the second in women (8.9/14.0 per 100,000 for rectal/colon cancer), as well as the fourth most common cause of cancer death [3,4]. The prevalent localization is the right colon (40%) followed by the left colon (31%) and the rectum (28%). Rectal cancer affects male patients slightly more often (31% versus 29% in women); also, men were found to be younger at the diagnosis (average age 63 versus 65 years for women) [1]. The 5-year survival ranges widely—from 5 to 93%—depending on the TNM stage of the disease, showing an average rate of 66.5% for rectal cancers compared to 64.2% for colon cancers [5,6].

According to the stage and the localization of the rectal tumor, we used the following radical surgical procedures: anterior rectal resection (ARR), low anterior rectal resection (LARR), very low anterior rectal resection (VLARR), Hartmann’s procedure (includes the preservation of the sphincter apparatus of the anus and a potentially reversible colostomy), and the abdominoperineal resection of the rectum (APR)—an irreversible procedure that involves the excision of the anus, the rectum, and a part of the sigmoid colon with adjacent lymph nodes through a double surgical approach (abdominal and perineal incision) and a permanent colostomy. The first three procedures include a colorectal anastomosis allowing a natural bowel transit; however, in some irradiated cases, a temporary (3–6 months) lateral protective colostomy or ileostomy was performed. All these surgical interventions made with a curative intent include either a total or a partial mesorectal excision (TME/PME) [7,8]. Low-lying rectal cancer (LRC) is defined as a malignant tumor located less than 6 cm (in some studies—5 cm) from the anal verge. Surgery is the main treatment option for resectable LRC and consists of APR. This type of surgery can be performed as a single treatment method or as a part of a multidisciplinary management protocol in combination with radiotherapy and chemotherapy as a neoadjuvant treatment or used postoperatively following the Oncological Committee decision issued for each case. Recently, the incidence of APR practice has reduced given the advances in radio- and chemotherapy, allowing the usage of ultralow anterior resection of the rectum in selected LRC cases [9,10,11,12].

Curative surgery is possible to cause significant perineal soft tissue defects that require reconstruction. Perineal reconstruction can be performed by direct closure, one of the basic options in the reconstructive ladder, up to musculocutaneous flaps and meshes (less frequently), and occasionally with adjuvant negative pressure wound therapy (NPWT) followed by skin grafting. Nowadays, the most used methods are direct closure and flap reconstruction [7,13,14]. In situations where the perineal defect created after the excision of the main tumor is too large and/or transfixiant and direct closure cannot be performed because of the high risk of complications, reconstruction with flaps is preferred. The prevention of perineal incisional hernia remains an important goal as well, thus requiring the initial reconstruction after APR to be performed using a tension-free surgical suture and thus limiting postoperative complications [7,8]. Multiple and varied options are currently available for perineal reconstruction; therefore, the challenge is to determine which type of flap is to be chosen. There is no consensus regarding the reconstructive method that should be adopted for perineal defects, and it has not been proven that “one flap fits all”. Due to the numerous reconstructive options available after APR, the development of decision-making algorithms for choosing the appropriate technique, based on clinical experience and the current literature data, can become a valuable tool for refining and personalizing the surgical management plan for each patient with perineal soft tissue defects [15,16].

## 2. Materials and Methods

The studied group of 245 patients who underwent curative surgery for LRC in the first Oncological Surgery Department of the Regional Institute of Oncology (IRO), Iasi, over 7.7 years (May 2012–December 2019) were identified. All subjects gave their informed consent for inclusion before they participated in the study. The study was conducted following the Declaration of Helsinki, and the protocol was approved by the Ethics Committee of the University of Medicine and Pharmacy “Grigore T. Popa”, Iasi, project approval no. 176/5.11.2019.

The study was conducted as follows: the identification of the patients from the Ist Oncological Surgery Clinic from the Iasi Regional Institute of Oncology with LLRCs treated by APR with the reconstruction of the defect by direct closure or reconstructive flaps in the period between May 2012 and December 2019; establishing the incidence of APR and the clinical and pathological characterization of the patients included in the study; and the development of customized decision algorithms for the reconstruction of pelvic/perineal defects, aiming to ease the choice from the wide range of possible reconstructive options, mainly based on flaps.

Inclusion criteria: patients over 18 years of age with lower ampullary rectal neoplasm in whom the surgical treatment involved APR; complete information record by studying the patient’s files related to the tracked data; and complete information regarding the multimodal treatment of patients: neoadjuvant or adjuvant therapy.

Exclusion criteria: other chosen surgical treatment except APR, incomplete medical data, and negative informed consent.

The patient’s medical data were collected from the clinical records, using the centralized IT system of the Regional Institute of Oncology Iasi—“Hipocrate”.

The recorded data were as follows: age and gender of the patients, the type and localization of the rectal neoplasm, diagnostic methods, type of curative surgery, methods of reconstruction after surgery, i.e., direct closure or reconstruction with flaps, the anatomopathological staging of the rectal tumor according to the AJCC and TNM classification, occurrence of complications and their resolution, and administered neoadjuvant and adjuvant treatments.

All the surgeries have been performed under general anesthesia with associated oro-tracheal intubation. All the excised tumors have been sent for histological examination. The indication for associated radio-chemotherapy has been discussed in a multidisciplinary committee (general surgery, plastic surgery, oncology, radiotherapy, and palliative care). The excision surgery was performed by a general surgery team and the flap reconstruction procedure by a plastic surgery team, respectively.

Analyzing each case of the studied group from the point of view of preoperative radiotherapy, intraoperative position, surgical technique, perineal defect volume, quality of tissues and perforators, and indications for direct closure or perineal reconstruction using flaps, flap type, and rate of postoperative complications, the definition of personalized algorithms was proposed.

## 3. Results

In the group of 245 patients with LLRC and treated using APR that was studied, 167 patients were male. The mean age was 63.33 ± 10.7 years with a mean of 64 years. Most patients (82.42%) were over 55 years of age, with the 60–69 age group (34.72%) being prevalent, followed by the 70–79 age group (30.4%). Cardiovascular comorbidities were predominant (53.55%), followed by metabolic (26.77%) (type II diabetes, obesity), urological (14.22%), and hepatic (8.36%) comorbidities.

Neoadjuvant radiotherapy was applied in 73.64% of patients and chemotherapy in 58.99% of cases. An extralevatorian variant of APR was performed in 99.16% (ELAPE) of patients, and the ischional technique was applied in 0.83% of cases; coccyx excision was required in 69.59% of patients. Immediate postoperative complications were minimal, less than 2%, with no infectious complications; among late complications, 12 cases (5.02%) were identified with perineal eventration. The indication for associated radio-chemotherapy has been discussed in a multidisciplinary committee (general surgery, plastic surgery, oncology, radiotherapy, and palliative care).

From all the patients of the studied group who underwent the APR procedure (245 cases), direct suturing was used to close the post-excisional soft tissue perineal defect in 239 cases (97.55%), while 6 cases (2.45%) benefited from the usage of various types of flap reconstruction techniques: 2 cases with vertical rectus abdominis muscle (VRAM) flaps, 2 cases with gracilis flaps, and 2 cases with gluteus maximus flaps.

Histopathological examination revealed, in many patients, the classic appearance of adenocarcinoma (ADK) (96.65%); at TNM staging, for stage T, pT3 predominated (55.64%), for stage N, pN0 predominated (56.90%), followed by pN1 (25.96%), and for stage M, 86.61% of patients were metastasis-free (M0). In patients with distant metastases, liver metastases were most common (8.36%), followed by pulmonary metastases (2.92%), peritoneal metastases (0.83%), and bone metastases (0.4%).

Clinical and pathological peculiarities of 239 patients (165 males, 74 females) who received LCR treated by APR with perineal defect reconstruction by direct closure technique of the perineal defect are revealed in the following Table 1.

Regarding the group of six patients with pelvic/perineal reconstruction involving flaps, the clinical characteristics and evolution presented by the 6 patients with pelvic/perineal reconstruction involving flaps were according to Table 2.

**Figure 1:**
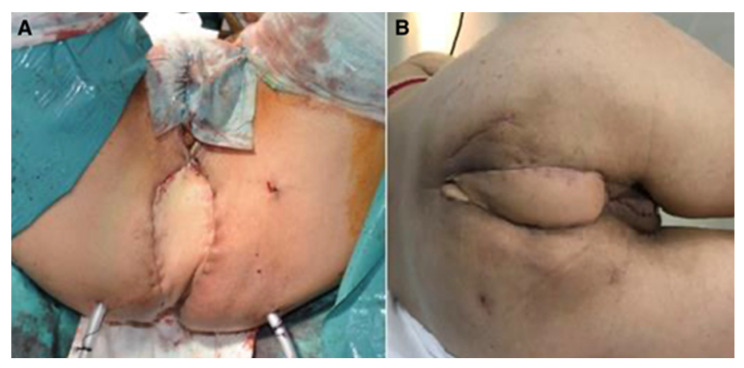
Perineal defect reconstructed with VRAM flap; (**A**)—immediate postoperative aspect; (**B**)—aspect after healing.

**Figure 2:**
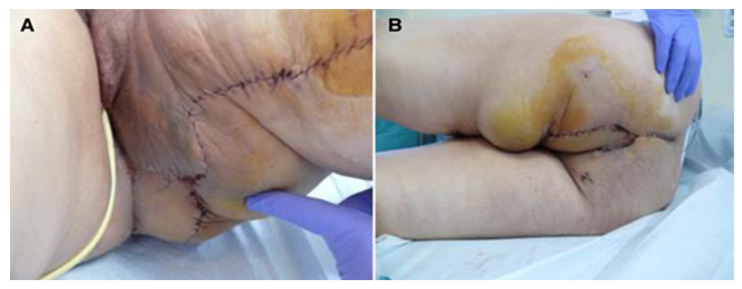
Perineal defect reconstructed with gracilis flap; (**A**)—direct suture of the donor site; (**B**)—the flap covering the soft tissue defect.

**Figure 3:**
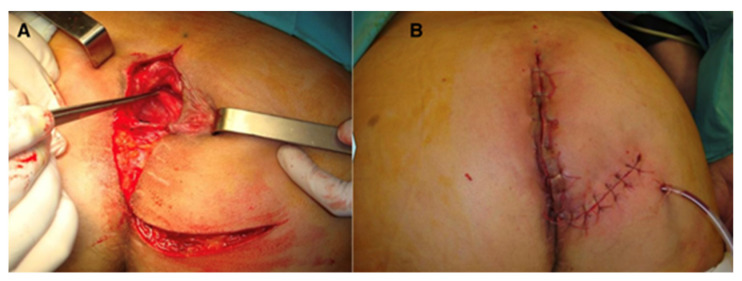
Perineal defect reconstructed with gluteus maximus flap; (**A**)—flap dissection; (**B**)—flap inset.

In the same group of patients (six cases), five cases received combined neoadjuvant radio- and chemotherapy. Among the comorbidities, we identified hypertension (three cases), anemia (two cases), and prostate adenoma (one case). Postoperative defects were large in all patients and did not allow direct closure; therefore, the option of choice was flap reconstruction; patients were followed for 17–72 months, with optimal flap healing, no intra- and postoperative complications, and no late complications (perineal eventration) (Figure 1, Figure 2 and Figure 3).

**Figure 4:**
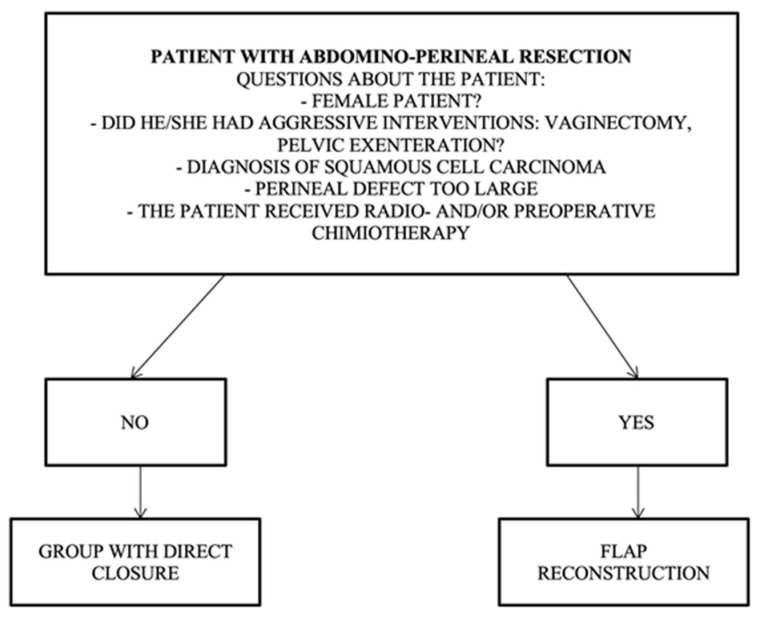
Algorithm for direct closure vs. flap reconstruction.

The patient profile with indications for direct closure should not contain any of the risk factors listed in the following algorithm: non-female patient, no aggressive curative intervention (vaginectomy, pelvic exenteration), no squamous cell carcinoma, a perineal defect that is too large, and no preoperative radio- and chemotherapy (Figure 4).

Patient profile for the group requiring flap reconstruction, and it should contain one of the following risk factors: female patient, aggressive surgery (vaginectomy, pelvic exenteration), diagnosis of squamous cell carcinoma, large perineal defect, preoperative radio/chemotherapy, and ventral positioning.

**Figure 5:**
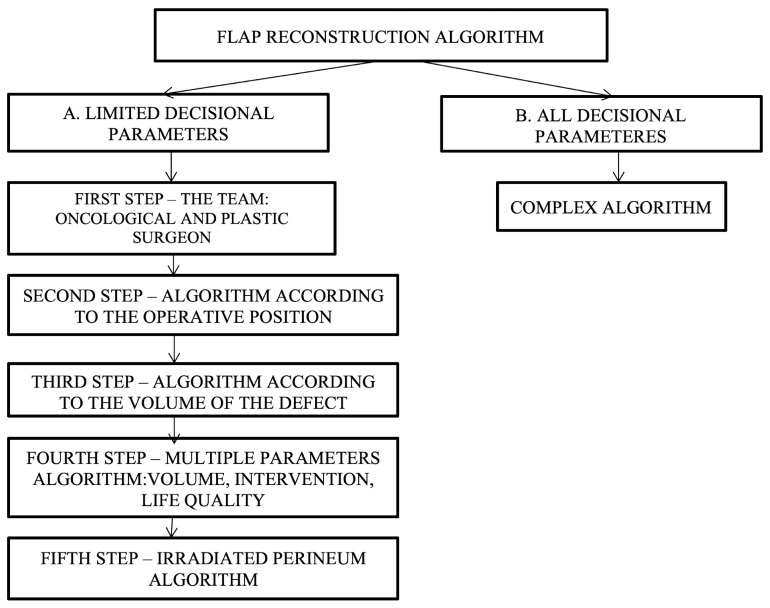
Decisional steps in developing and choosing the appropriate flap reconstruction algorithm depending on various parameters.

Since there are varied and multiple options available for reconstruction, specifying which type of flap to choose is a challenge. In developing decision algorithms, we set out to go through this process in several stages, i.e., decision steps, as follows: (a) the development of simple algorithms with a limited number of decision parameters and (b) the development of a complex algorithm—all decision parameters (Figure 5).

**Figure 6:**
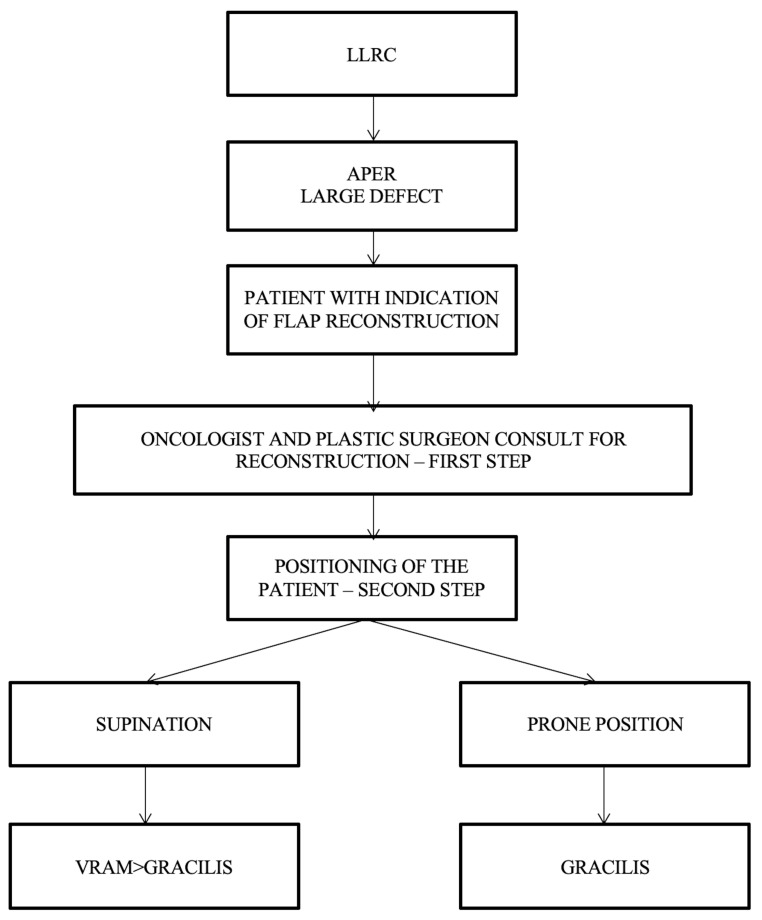
Algorithm according to the patient’s intraoperative position. LLRC = low-lying rectal cancer; APER = abdominoperineal excision of the rectum; and VRAM flap = vertical rectus abdominis muscle flap.

Simple algorithms with a limited number of decision parameters refer to the cases in which the oncologic surgeon cannot resolve the perineal defect by direct closure, and a plastic surgeon should be consulted. After consultation between the oncological surgeon and the plastic surgeon, the next step consists of establishing the patient’s intraoperative position, which can be decided using the following proposed algorithm (Figure 6).

There are no definitive management recommendations for perineal defects after APR. Each flap and its variants have advantages and disadvantages. Depending on the donor sites, flaps can be listed, as follows. Abdominal flaps: VRAM (vertical rectus abdominis myocutaneous flap); MsVRAM (muscle-sparing vertical rectus abdominis myocutaneous flap); FsVRAM (fascia-sparing vertical rectus abdominis myocutaneous flap); and DIEP (deep inferior epigastric perforator flap). Gluteal flaps: IGAP/IGAP (superior or inferior gluteal artery perforator flap), IGAM (inferior gluteal artery musculocutaneous flap), V-Y perforator flap, and V-Y advanced fasciocutaneous flap. Pudendal flap; IPAP (internal pudendal artery perforator flap), gluteal fold flap (GFF), lotus petal flap, Singapore flap, thigh flaps, gracilis flap, posterior thigh flap, and anterolateral thigh flap (ALT).

For the patients operated in a supine/lithotomy position, the VRAM flap is the most popular choice of reconstructive surgery after APR and is indicated in patients with large defect volume, over 60 sqcm, and for those with pelvic exenteration or sacral bone resection, after which a large volume of dead space remains. The right rectus abdominis muscle is preferred as the colostomy passes through the fibers of the left muscle (the technique used in our cases allows the intermittent evacuation of the feces in the colostomy bag). In cases receiving surgery in a prone position, the gracilis flap is recommended because the VRAM flap is not accessible. The gracilis flap is considered an efficient option, as it offers a large arc of rotation, no movement restrictions, and posterior ambulation. As a disadvantage, the gracilis flap may be smaller in size and is not used for larger pelvic/perineal defects.

**Figure 7:**
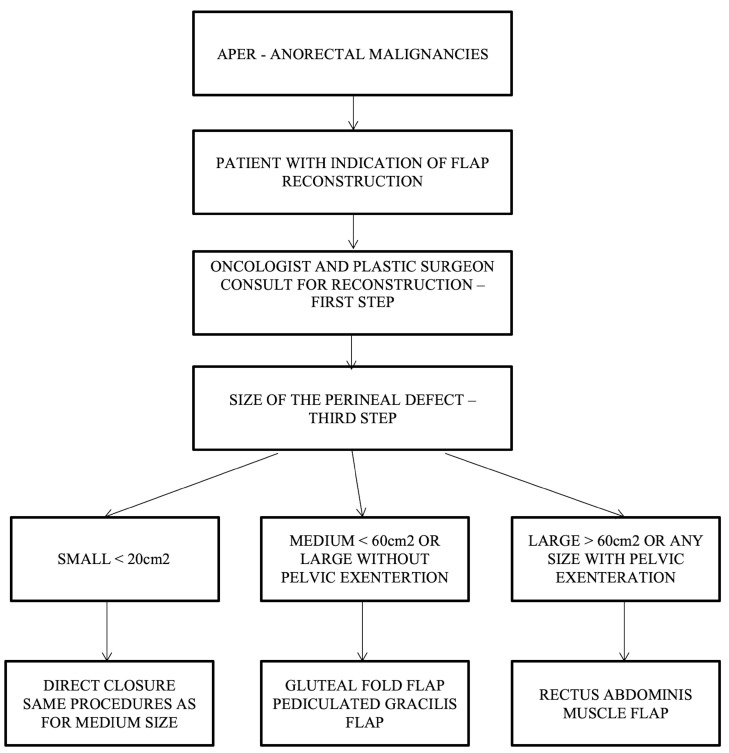
Algorithm for choosing the type of flap in perineal reconstruction after APR following the defect’s volume.

Our team proposes a surgical management algorithm according to the defect’s volume, as presented in (Figure 7).

**Figure 8:**
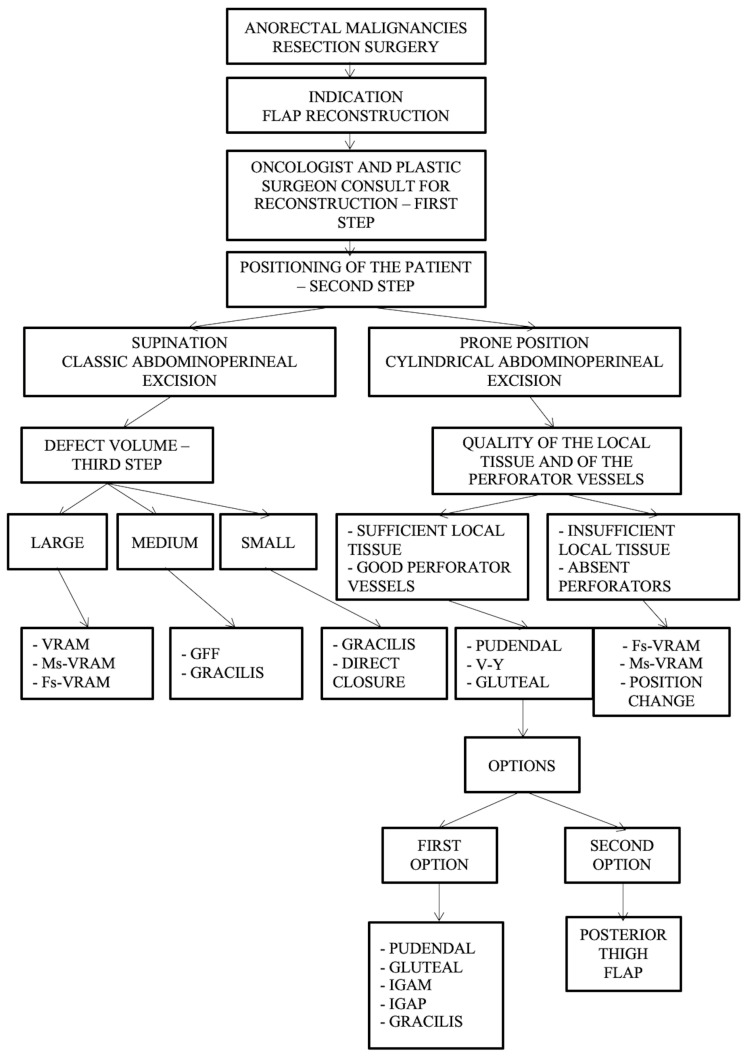
Algorithm according to intraoperative positioning, the type of intervention, defect volume, quality of the local tissue, and perforator vessels. Abdominal flaps: VRAM (vertical rectus abdominis myocutaneous flap); MsVRAM (muscle-sparing vertical rectus abdominis myocutaneous flap); FsVRAM (fascia-sparing vertical rectus abdominis myocutaneous flap); and DIEP (deep inferior epigastric perforator flap). Gluteal flaps: IGAP/IGAP (superior or inferior gluteal artery perforator flap), IGAM (inferior gluteal artery musculocutaneous flap), V-Y perforator flap, V-Y advanced fasciocutaneous flap, and gluteal fold flap (GFF).

Furthermore, we have developed a complex management algorithm according to patient position, type of intervention, volume, and quality of local tissue and perforators as presented in Figure 8.

Important aspects of the presented algorithm that should be highlighted:

- The VRAM flap is preferred for large defects.
- If the patient has an old midline scar if he/she has optimal CT or Doppler-investigated perforators, MsVRAM or FsVRAM is recommended.
- MsVRAM is a fast, easy-to-harvest flap that allows safe and fast reconstruction with fewer sequelae in the context of laparotomy, also avoiding the abdominal wall sequelae of the conventional VRAM flap as sufficient muscle and fascia remain available for reconstruction.
- In the prone position, perforator-based flaps based on the internal pudendal artery with gluteal or pudendal donor site are preferred. SGAP/IGAP gluteal flaps, IGAM flaps, and advanced fasciocutaneous V-Y flaps, or perforator-based, as well as pudendal flaps such as GFF, lotus petal flaps, or Singapore flaps, can be used.
- The choice of the optimal reconstructive method must be initially motivated by the imperatives of surgical excision.

Reconstruction algorithm for preoperatively irradiated perineum.

For patients who have received radiotherapy, flaps with a non-irradiated donor site, distant from the perineal region, such as the VRAM flap, gracilis flap, gluteal fold flap, IGAP/IGAM flap, or pudendal thigh flap, are used; currently, the VRAM and gracilis flap are in focus. These flaps have the advantage of providing healthy, well-vascularized tissue and offer both an optimal muscle volume to fill the pelvic dead space and the possibility of vaginal reconstruction if necessary.

**Figure 9:**
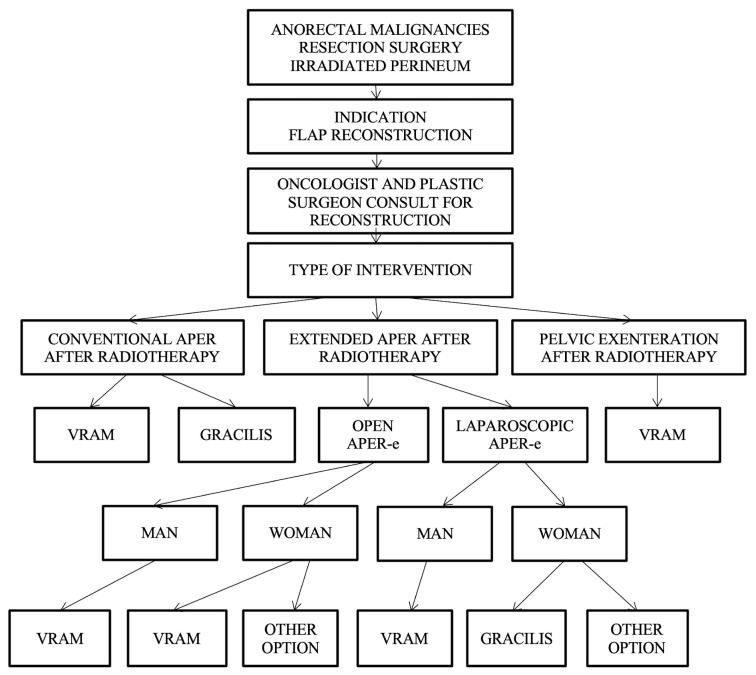
Algorithm for the reconstruction of preoperative irradiated perineum.

Taking the patient’s history of perineal irradiation into account as a decisional factor, the VRAM flap, gracilis, and posterior thigh flap are strongly recommended; the gluteal and pudendal flaps are suggested as possible options. The algorithm consider the irradiation of the perineum as well as the type of curative intervention in choosing the optimal reconstructive technique (Figure 9).

The algorithm initially requires the presence of a patient with anorectal malignancies treated with APR, followed by the indication for flap reconstruction and by a consultation between the general surgeon and the plastic surgeon.

Depending on the type of surgery, which can be conventional APR without radiotherapy, open extended APR, or laparoscopic or pelvic exenteration, the choice of the flap is made as follows:

- Conventional APR with irradiated perineum—VRAM flap or gracilis flap are recommended.
- Extended APR with irradiated perineum, and in which case, two situations exist:
  - Conventional open extended APR, case in which the VRAM flap is recommended in both men and women or possibly other options in women (uterine retroversion).
  - Laparoscopic extended APR is the case in which the gracilis flap is recommended for men, and the gracilis flap or other options are recommended for women.
- Pelvic exenteration—VRAM flap is recommended.

**Figure 10:**
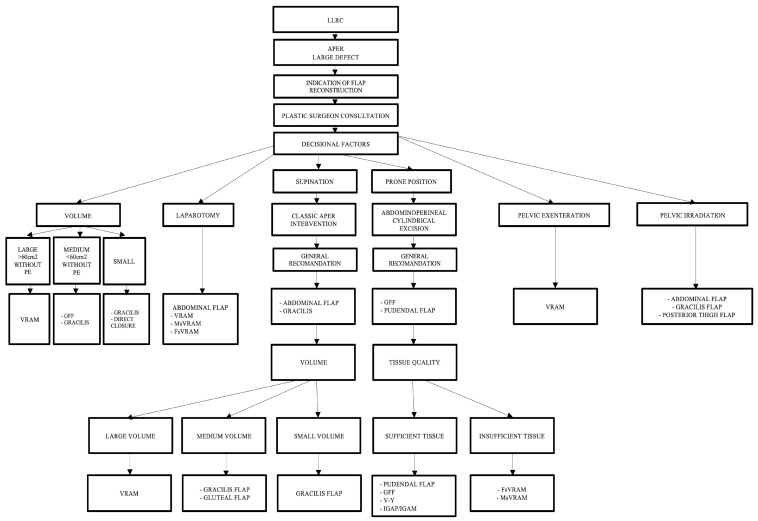
Complex algorithm including all decision parameters.

The algorithm-based approach is useful for the practicing general surgeon, oncologist, or plastic surgeon, as it allows for relevant personalized decisions to be made, followed by a decrease in the rate of usual complications that can occur following the reconstruction of these irradiated perineal defects, as well as a reduction in care costs. The complex algorithm that includes all decision parameters is presented in Figure 10.

## 4. Discussion

The main objectives of our study were to establish the clinical and pathological profiles of patients with LRC undergoing APR and to use these multifactorial data to initially develop or improve the few pre-existing decision algorithms in the literature for choosing the most appropriate surgical procedure to reconstruct the soft tissue perineal defects ranging from direct closure to flap reconstruction [7,17,18,19,20,21,22].

In our study, most patients underwent direct closure of the defect (97.55%) and the remaining 2.45% benefited from flap reconstruction. Direct closure reconstruction is the most common way to repair the perineal defect after APR, although, as described in the literature, it is an approach associated with a high local complication rate of 41% (ranges from 35 to 66% as perineal wound infection, dehiscence or perineal incisional hernia), especially when high suture tension exists. Choosing this procedure requires assessing and avoiding risk factors such as wound tension, pelvic–perineal defects with a very large dead space volumes, large or very large perineal wounds, insufficient drainage possibilities, preoperative neoadjuvant radiotherapy, comorbidities, or aggressive techniques [16,23,24,25,26,27,28,29].

Contrary to what has been reported in this field, in the studied series of cases, the complication rate was low. No immediate postoperative infectious complications of midline perineal wounds or pelvic abscesses were reported. Although it is claimed that patients who receive radiotherapy in combination with the extralevatorian abdominoperineal excision of the rectum (ELAPE) have more issues with the perineal defect, in our patients, direct closure was possible without complications. Even though a significant percentage of cases received radiotherapy (73.64%) and chemotherapy (58.4%), it was assessed that the pelvic dead space was not too large, being sufficient, and effective drainage was performed, and the wound did not have significant tension after closure. In six patients, flap reconstruction was performed. In some studies, with large series of patients, primary (direct) closure was performed in more than 50% of cases and flap closure in 21–24% of cases, although some authors mention higher percentages (86%) for flaps [7,30,31]. Complex cases that underwent aggressive surgeries, such as those who received ischional variant of APR, usually present important perineal defects and a large pelvic “dead space”—a potential collection area with risk of infection and intestinal occlusion due to the adherences developed in the so-called empty pelvis syndrome. These cavities (three-dimensional pelvic dead space) must be filled as much as possible using reconstructive techniques using flaps that warrant consultation with the plastic surgeon [17,18,32].

The optimal reconstructive option in the studied patients was the use of musculocutaneous flaps, with the VRAM (two cases), gracilis (two cases), and gluteus maximus (two cases) flaps being preferred. The VRAM flap is recommended as a first-line option by considering the advantages and disadvantages for each patient [33,34,35]. The gracilis flap is indicated in perineal reconstruction for small, superficial defects and is not recommended for large, wide defects in the perineum [36]. The gluteus maximus muscle flap may be an option for reconstruction but is burdened with functional static disorders; thus, it is less commonly used [37,38].

In the cases observed during the study, there were no such functional disorders. A commonly used option in practice is the gluteal fold flap (GFF), which may be an ap-propriate option for the reconstruction of mid-perineal defects and some large defects after APE, being a safe, robust flap, but which has not been practiced by us. The question is as follows: which flap should be used? Due to the numerous reconstructive options available after APR, based on clinical experience and published literature data, we have developed a series of decision algorithms for choosing the appropriate, customized technique with the objective of simplifying the decision-making process [34,39].

Algorithm sequencing has been proposed in the form of decision steps from simple to complex. Simple algorithms with limited decision factors (patient position, defect volume, and tissue quality) or more complicated algorithms with multiple decision factors (operative position, defect volume, type of surgery, tissue and perforator quality, and perineal irradiation) have been developed to facilitate the planning of the optimal reconstructive surgical technique for the perineum. Finally, a comprehensive algorithm has been developed that includes all decision factors to create a framework guideline for oncological and plastic surgeons involved in this field. The elaboration of the preoperative plan in a multidisciplinary team is essential to obtain a good optimal result. It was insisted that the gastrointestinal surgeon must “team-up” with the plastic surgeon, as “step I”, to which the participation of other specialists (radiologist, anesthesiologist, oncologist, pathologist, and palliative care) is added, to develop the best working strategy. Given this wide range of reconstructive knowledge, as well as the rich arsenal of surgical techniques for reconstruction, plastic surgeons must answer the question “Which flap, where, when, and how?”. Algorithms can represent tools for plastic surgeons as well as oncological surgeons in pelvic reconstruction, and they are helpful in surgical decision making. The algorithms presented provide solutions for solving perineal wound reconstruction after APR and are responsible for the choice of the reconstructive technique [17,36,37,38]. In the recent specialized literature, there is a limited number of authors who are preoccupied with the issue of perineal reconstruction and who have designed various algorithms based on different visions and experiences. The algorithms created by us include a complete and balanced analysis of all the factors involved in the decision-making process. In their development, we have taken into consideration both our own case management experience and the algorithm models developed by Sheckter, C.C. et al. [19], John, H.E. et al. [13], Sinna, R. et al. [40], and Saleh, D.B. et al. [41].

However, we consider that a larger study is needed in the future to confirm the efficiency of our management plan. Decisional algorithms have the potential to represent a valuable guideline in the potential development of national or international treatment protocols.

The limitations of the study consisted of the relatively small number of patients who required the reconstruction of post-excisional soft tissue defects with flaps.

## 5. Conclusions

The management of perineal soft tissue defects post-APR depends on patient-related features, surgery-related factors, and defect characteristics. Given the lack of consensus regarding the optimal reconstructive method, we have proposed a series of decision algorithms, based on clinical experience and literature data, to simplify the decision-making process. These algorithms range from simple ones with limited decision factors to more complex ones with multiple decision factors, culminating in a comprehensive algorithm that includes all decision factors. Developing the surgical plan within a multidisciplinary team, especially including plastic surgeons, is essential for optimal outcomes. Plastic surgeons must address the questions of “Which flap, when, who, and how?” The proposed decisional algorithms may be found useful in making efficient complex surgical decisions and can serve as potential guidelines for designing specific protocols, but their efficiency should be confirmed in a larger, metanalytical type of study.

## Figures and Tables

**Table 1:** Basic studied parameters of the group with perineal defect reconstruction by direct closure.

Parameter	Number	%
**Age (Years)**	63.33 +/− 10.7	
**Sex**		
M	165	69.03
F	74	30.96
**Neoadjuvant** **Radiotherapy**		
+	176	73.64
−	63	26.35
**Neoadjuvant ** **Chemotherapy**		
+	141	59.99
−	98	41.00
**Abdominoperineal ** **Resection**		
- EXTRALEVATORIAN	237	99.16
- ISCHIONAL	2	0.83
**Coccyx Excision**		
+	152	63.59
−	87	36.40
**T Stage**		
pT0	7	2.92
pT1	10	4.18
pT2	70	29.28
pT3	133	55.64
pT4	19	7.94
**N Stage**		
N0	136	56.90
N1	62	25.94
N2	41	17.15
**M Stage**		
M0	207	63.61
M1	32	13.38
**AJCC TNM stage**		
p CR	7	2.92
0	68	28.45
I	46	28.45
II	82	34.30
III	37	15.48
IV	4	1.67

AJCC = American Joint Committee on Cancer.

**Table 2:** Parameters of the patients who underwent flap reconstruction after APR.

Patient	Sex	Age	Histological Result	Indications	Preoperative Radiotherapy	Preoperative Chemotherapy	Mesorectum	Comorbidities	Metastases	Curative Surgery	Reconstruction	Results, Evolution
1.	F	57	ADK	large volume, ELAPE	+	+	G2	HTA	M0	ELAPE	VRAM	good
2.	F	53	ADK	large volume, ELAPE	+	+	G2	anemia	M0	ELAPE	Gracilis	good
3.	M	63	ADK	large volume, ELAPE	−	−	G2	anemia	M1	ELAPE	Gluteus	good
4.	F	68	ADK	large volume, ELAPE	+	+	G3	-	M0	ELAPE	Gracilis	good
5.	M	74	ADK undiff.	large volume, ischional APR	+	+	G2	HTA, prostate adenoma	M0	ischional APR	VRAM	good
6.	F	73	ADK	large volume	+	+	G3	HTA	M0	ELAPE	Gluteus	good

ADK = adenocarcinoma; APR = abdominoperineal resection; ELAPE = extralevatorian abdominoperineal excision; and VRAM flap = vertical rectus abdominis muscle flap.

## Data Availability

Data are contained within the article.
